# A reference interval study of serum 25-Hydroxyvitamin D among an African elderly population

**DOI:** 10.4314/ahs.v22i4.27

**Published:** 2022-12

**Authors:** Ifeyinwa Dorothy Nnakenyi, Ezra Ogbonnaya Agbo, Emeka Francis Nnakenyi, Victor Chukwuma Wakwe

**Affiliations:** 1 Department of Chemical Pathology, University of Nigeria Teaching Hospital, Ituku/ozalla, Enugu, Nigeria; 2 Department of Morbid Anatomy, University of Nigeria Teaching Hospital, Enugu, Nigeria; 3 Department of Chemical Pathology, University of Port-Harcourt Teaching Hospital, Port-Harcourt, River's state, Nigeria

**Keywords:** Bootstrap method, elderly, reference interval, vitamin D

## Abstract

**Background:**

Elderly people have increased risk factors for low serum vitamin D levels, which is worsened among the black race. Therefore, elderly Africans constitute a reference population for vitamin D study.

**Aim:**

The aim of this study was to establish the reference interval of serum 25-hydroxyvitamin D (25(OH)D) among an African elderly population.

**Methodology:**

This was a cross-sectional study of rural community dwellers in Enugu, south-eastern Nigeria aged 50 years and above, that satisfied the criteria of the reference population. Ethical approval and informed consent were obtained. Venous blood was collected from reference individuals and serum 25(OH)D was determined by enzyme-linked immunosorbent assay. Data were analysed using a non-parametric, bootstrap method to obtain the central 95% reference limits and 90% confidence intervals of the lower and upper limits of the reference interval respectively.

**Results:**

One hundred and twenty-four (62 males and 62 females) participants were recruited. The median (25th -75th percentile) of serum 25(OH)D was 56 (35 – 71) ng/ml. The 2.5th percentile defined the lower reference limit and it was 21 ng/ml with 90% confidence interval (20 – 23) ng/ml; while the 97.5th percentile defined the upper reference limit and it was 93 ng/ml with 90% confidence interval (90 – 98) ng/ml.

**Conclusion:**

The reference interval for serum 25(OH)D for the selected African elderly population in Enugu, Nigeria was determined to be 21 to 93 ng/ml.

## Introduction

Vitamin D is a fat-soluble vitamin that has two major metabolites in humans: ergocalciferol (D2) and cholecalciferol (D3). Both forms contribute to the overall vitamin D status.^1^ Vitamin D is 25-hydroxylated at the liver to form 25-hydroxyvitamin D (25(OH)D) and then, activated to 1,25-dihydroxyvitamin D (1,25(OH)2D) by the kidneys through the action of 1α-hydroxylase.^2^ This 1,25(OH)2D is the form in which vitamin D exerts its physiological roles, but the 25(OH)D form is 1000 times that of the former in serum, and this excess concentration constitutes a large storage facility.^1^ Therefore, vitamin D status is most accurately reflected by serum 25(OH)D concentrations.

Although vitamin D plays an important role in calcium, magnesium and phosphate metabolism, recent studies have shown that it is implicated in several other biological effects such as: cancer, diabetes mellitus, hypertension and cardiovascular diseases, multiple sclerosis, cognitive impairment and other psychological illnesses, amongst others.^3^ – ^7^ It has been found that virtually all the cells of the body has vitamin D receptors, which the vitamin interacts with to bring about transcriptional processes that modulate gene expression.^8^ The pathogenesis of some of these medical conditions involves a direct deficiency of vitamin D, a defect in the pathway/tissues involved in vitamin D metabolism or cases in which vitamin D insufficiencies have been noted.^9^ Ageing also affects the overall processes of vitamin D metabolism in the body.

The elderly are more prone to develop vitamin D deficiency because of various risk factors which include: decreased dietary intake, diminished sunlight exposure, reduced skin thickness, impairment of intestinal absorption and hydroxylation in the liver and kidneys.^9^,^10^ There is also a slowing down in the biochemical processes involved in vitamin D metabolism. All these affect the bioavailability of vitamin D. 9 Therefore, the elderly forms a unique population for vitamin D study.

A clinical laboratory test result is interpreted with an appropriate reference interval. Based on different preanalytical variables such as age, it is important to establish reference intervals for particular populations. Reference intervals are typically established by testing a large number of healthy population and determining what appears to be “normal” for them.^11^ A critical step in establishing reference interval is to define the reference population. A reference population is defined as a representative sample of individuals used to establish the norm for reference intervals.^12^ It represents a universal set from which representative samples (subsets) are obtained for study. Demographically, it should match the population whose laboratory results will be compared to this reference interval.^12^ Globally, the increase in life expectancy due to advancement in medical sciences leading to decline in old age-related mortality has led to an increase in the number of aged population.^13^ If the trend should continue it is projected that by 2050, the number of individuals older than 60 years will be approximately 2 billion and will account for 22% of the world's population.^14^ Similarly, in Nigeria, a large fraction of the country's population is made up of elderly people.^10^ In view of this growing population of elderly people, a study to establish a local reference interval of serum 25(OH)D for this reference population is timely.

Although Nigeria has a tropical climate, which has a long seasonal duration of sunny days, studies have shown that dark-coloured skin (which contains more melanin) synthesizes vitamin D poorly,^15^ and has resulted in the increased prevalence of vitamin D deficiency using foreign derived reference intervals and criteria.^16^ As a result, elderly people in Enugu, south-eastern Nigeria, constitute a reference population for vitamin D study. Therefore, we aimed to establish a reference interval for serum 25-hydroxyvitamin D among an African elderly population, in order to prevent overestimation of low vitamin D status among them.

## Method

The study was a descriptive cross-sectional study of serum levels of 25-hydroxyvitamin D (25(OH)D) to establish a reference interval for an African elderly population using a direct approach.

### Ethical considerations

Ethical clearance was obtained from the Human Research Ethics Committee (HREC) of the University of Nigeria Teaching Hospital (UNTH) Enugu (NHREC/05/01/2008B-FWA00002458-1RB00002323) before commencement of the study and permission sought from community leaders of the communities that were involved in the research. Also, informed consent was obtained from each participant before enrolment into the study. Confidentiality of participants' information was ensured.

### Study Population

The participants were recruited from three rural communities of Enugu state, southeast Nigeria: Ituku community in Awgu Local Government Area (LGA), as well as Akpakume and Nze communities both in Udi LGA. Ituku community is located at about 5km southwest of the state capital at about latitude 6048′ NE. Akpakume and Nze communities are about 9km northwest of the state capital at about latitude 7012′ NE.^17^ These communities are predominantly made up of elderly people as their youths have migrated to the city. The weather conditions there consist of rainy and dry seasons. The rainy season is usually from March to August while dry season is from September to February annually. The average daylight hours per day is 12hours.^18^ Community dwellers in these places are mainly subsistence farmers and stay out-doors for most time of the day and so have adequate exposure to sunlight. However, their foods consist mainly of cassava, yams and rice, which are poor in vitamin D. There is also no river in and around these communities and so diets are poor in sea-foods, which are rich in vitamin D.

### Reference Population

A medical outreach was conducted during the dry season (November) to recruit individuals for this study. According to the United Nations (UN), the elderly refers to people aged 60 years and above.^19^ However, the World Health Organization (WHO) proposed 50 years and above as definition of an elderly person in Africa due to relatively lower life expectancy and smaller size of older population in the region.^20^ Therefore, our inclusion criteria were apparently healthy persons aged 50 years and above, who had normal calcium metabolism evidenced by the serum albumin-adjusted calcium concentrations within 2.15–2.55 mmol/l. Those excluded were hypertensive patients (anyone with blood pressure greater than 140/90 mmHg or on anti-hypertensive medication), anyone with renal disease (estimated glomerular filtration rate less than 60 ml/min/1.73m^2^), diabetic mellitus patients (random blood glucose greater than 11.1 mmol/l or on anti-diabetic medication), anyone on vitamin D supplementation, anyone with any musculoskeletal disease e.g. history of falls, fractures and arthritis, and anyone with stroke or any other known illness (as was obtained from the clinical assessment and questionnaire).^21^

Questionnaires were administered by an interviewer to obtain information about the biodata, medical and drug history. In order to exclude those who were hypertensive, the blood pressure of the participants was measured by auscultation method after they had rested for 10 minutes. Their random blood glucose was determined using glucometer (Accu-Chek® USA) in order to exclude diabetes mellitus patients. Serum creatinine was used to determine estimated glomerular filtration rate and then exclude those with renal disease. Then a general clinical examination was conducted to exclude those with any other obvious illness including liver disease.

### Reference Individuals

The reference individuals were selected from the reference population and partitioned into subgroups based on age in years as follow: Group I: 50–59; Group II: 60–69; Group III: 70–79; and Group IV: 80 and above. We targeted a minimum sample size of 120 reference individuals, which is optimal for reference interval studies.^22^ An extra 30% was included to cater for attrition loss and undesired laboratory results (blood calcium, glucose and eGFR). In total, 200 participants were sampled.

### Specimen Collection and Processing

Five (5) ml of venous blood was aseptically collected via venipuncture into a plain bottle. They were transported to the Department of Chemical Pathology, University of Nigeria Teaching Hospital (UNTH), Ituku-Ozalla, a tertiary health institution in Enugu, south-eastern Nigeria for processing and analyses. The blood specimen was allowed to clot and retract by allowing it to stand for 45 minutes, after which it was centrifuged at 4000 rpm for 10 minutes. Then, the serum supernatant was aliquoted into two cryovials for measurements of 25(OH)D, as well as calcium, albumin and creatinine concentrations. Sera were stored frozen at -20°C until analyses were performed. Repeated freeze-thaw cycles were avoided.

### Specimen analyses

Participants' serum calcium and albumin concentrations were determined using Arsenazo-III and Bromocresol Green spectrophotometric methods respectively^23^,^24^, and then albumin-adjusted calcium concentration was calculated in order to exclude those with abnormal calcium values. Also, serum creatinine concentrations were determined using Jaffe kinetic method^25^, and estimated glomerular filtration rate (eGFR) was calculated using chronic kidney disease - epidemiology collaboration (CKD-EPI) equation in order to exclude those with renal diseases.

### Reference Values

The serum 25(OH)D concentrations were determined using a solid-phase enzyme-linked immunosorbent assay (ELISA) (Calbiotech®, USA) to obtain the reference values. This assay had an analytical sensitivity of 1.25ng/ml, with a measurable range up to 150ng/ml. 26 The intra-assay coefficient of variation was <6%, while the total imprecision was <8%.^26^ This assay had standards that were traceable to NIST SRM-972A, and showed excellent correlation to LC/MS/MS (n=20, R2 = 0.949).^26^ Analytical accuracy and precision was ensured during analyses using commercially prepared control sera (Calbiotech®, USA).

### Statistical analyses

The data obtained were analysed using Reference Value Advisor software on Microsoft Excel 2013.^27^

### Reference Distribution

Normality of the reference values (serum 25(OH)D concentrations) was determined using Anderson-Darling test. The serum 25(OH)D concentrations were sorted in ascending order and ranked consecutively. Outliers were detected using Dixon, as well as Tukey methods. The data were presented with a histogram and graph to show the reference distribution curve.

### Reference Limits and Interval

The reference interval was calculated using bootstrap, non-parametric method.^28^ The bootstrap method involved random resampling with replacement to obtain a large number of resamples.^29^ For each resample, the upper and lower reference limits of the 95% reference interval have rank numbers equal to 0.025(n + 1) and 0.975(n +1), which corresponds to the 2.5th and 97.5th percentiles respectively.^28^ The median of all the resample estimates at the 2.5^th^ and 97.5th percentiles defined the lower and upper reference limits respectively.^28^ The 2.5th to the 97.5^th^ percentiles were computed to obtain the central 95% reference interval.^22^,^30^ The 90% confidence intervals of each limit of the reference interval were also determined.^28^,^30^

Mann-Whitney U-test was used to compare median concentrations of serum 25(OH)D between the male and female subgroups. A ρ-value of < 0.05 was considered to be statistically significant.

## Results

A total of 124 participants (62 males and 62 females) were recruited as reference individuals from the initially enrolled population of 200 (62%). The mean ± standard deviation of their age was 70 ± 13 years. Their demographic and biochemical parameters are given in Table 1. The females were older than the males and this was statistically significant (p <0.05).

**Table 1:** Demographic and Biochemical Parameters of The Reference Individuals

Parameter (unit)	All subjects	Male	Female	p-value
Sample size (n)	124	62	62	
Age (years)	70 ± 13	66 ± 17	73 ± 16	0.01*
25(OH)D (ng/ml) #	56 (35 – 71)	54 (35 – 68)	56 (35 – 72)	0.35
Systolic blood pressure (mmHg)	133 ±7	139 ± 7	132 ± 5	0.53
Diastolic blood pressure (mmHg)	84 ± 5	86 ±7	82 ± 5	0.04*
Random blood glucose (mmol/l)	6 ± 1	6 ± 2	6 ± 1	0.33
Creatinine (µmol/l)	142 ± 9	149 ± 11	138 ± 9	0.04*
Estimated glomerular filtration rate (eGFR) (mL/min/1.73m^2^)	108 ± 9	96 ± 7	110 ±10	0.06
Calcium (mmol/l)	2.4 ± 0.1	2.3 ± 0.2	2.3 ± 0.1	0.09

# Serum 25(OH)D concentrations are expressed as median (25^th^ – 75^th^ percentile) while results of other parameters are expressed as mean ± standard deviation.

* Statistically significant difference (p<0.05).

**Figure 1:**
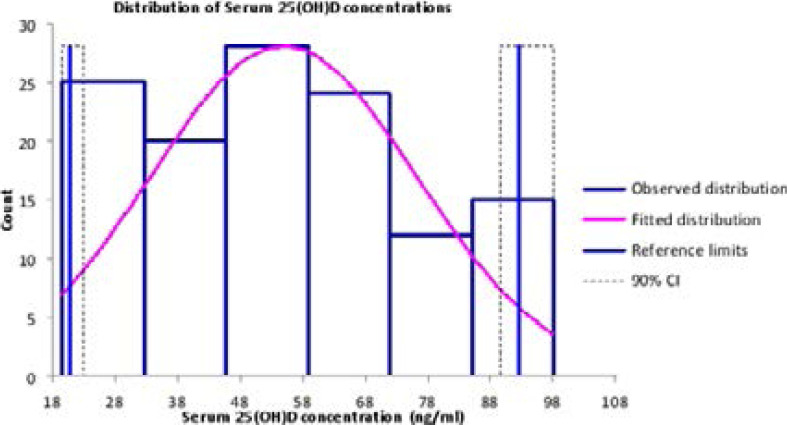
Distribution of serum 25(OH)D concentrations (ng/mL) of the elderly reference individuals

The reference values of the serum 25(OH)D concentrations gave a non-parametric distribution (Figure 1). The median (25th – 75th percentile) serum 25(OH)D concentration was 56 (35 – 71) ng/ml for all participants; 54 (35 – 68) ng/ml for male participants and 56 (35 – 72) ng/ml for female participants. There was no statistically significant difference between the 25(OH)D levels of the male and female participants, even after adjusting for age (p >0.05). The serum 25(OH)D concentrations declined with increase in age-group (Table 2).

**Table 2:** Serum 25(Oh)D Concentration of Different Age Groups Among the Reference Individuals

Group	Age range (years)	Sample size	25(OH)D (ng/ml) #
I	50 – 59	32	74 (60 – 88)
II	60 – 69	32	67 (57 – 75)
III	70 – 79	32	46 (36 – 55)
IV	80 and above	28	27 (23 – 32)
All subjects (Combined group)	≥50	124	56 (35 – 71)

# 25(OH)D levels were expressed as median (25^th^ – 75^th^ percentile).

Using the bootstrap method, 1000 random samples were generated. The 2.5th percentile was determined as 21 ng/ml with 90% confidence interval (20–23) ng/ml; while the 97.5^th^ percentile was determined as 93 ng/ml with 90% confidence interval (90–98) ng/ml. Likewise the 2.5^th^ and 97.5^th^ percentiles, with their respective 90% confidence intervals, were determined for each age group (Table 3). Therefore, the reference interval of this black African, elderly population was determined as 21–93 ng/ml.

**Table 3:** Percentiles, Reference Intervals and Median Serum 25(OH)D For the Different Age Groups of Reference Individuals

Group	Age range (years)	Sample size	2.5^th^ percentile (ng/ml)	90% CI of 2.5^th^ Percentile	97.5^th^ percentile (ng/ml)	90% CI of 97.5^th^ percentile	Reference intervals (ng/ml)	Median 25(OH)D (ng/ml)
**I**	50–59	32	54	47 – 60	94	87 – 101	54 – 94	74
**II**	60–69	32	38	31 – 45	97	90 – 104	38 – 97	67
**III**	70–79	32	30	23 – 35	62	55 – 68	30 – 62	46
**IV**	80 and above	28	20	16 – 23	35	32 – 39	20 – 35	27
**Combined** **group (All** **subjects)**	50–103 (≥50)	124	21	16 – 26	93	87 – 98	21 – 93	56

## Discussion

A reference population is defined as a study population whose members experience a similar disease (or physiological condition).^31^ Elderly people have experienced a similar physiological condition due to the effect of ageing on virtually all the structures and functions of their body tissues/cells. A reference interval provides useful information for interpretation of clinical laboratory results. Age-specific reference interval becomes more important as it further integrates the effect of physiological changes associated with that particular age group into the interpretation of such biochemical parameter.

We observed that serum 25(OH)D levels decreased with advancing age (Table 2). This is supported by a large study of 8042 individuals in Greece that reported a decline of 25(OH)D concentration with increase in age.^32^ This is due to several risk factors the elderly are prone to that affect their vitamin D metabolism.

From this study, the reference interval of serum 25-hydroxyvitamin D (25(OH)D) for the elderly was determined as 21–93 ng/ml. This interval satisfies the recommendation by Institute of Medicine (IOM) that serum 25(OH)D concentration of 20 ng/ml or more is sufficient. 33 But our lower reference limit is less than that recommended by the United States (US) Endocrine Society, which has a desirable limit of greater than 30 ng/ml^34^, as well as that by the US vitamin D Council which is 40–80 ng/ml^35^ (Table 4). The implication of this is that if the latter reference ranges were used in our population, we would have a higher prevalence of vitamin D deficient individuals. A study conducted in United Arab Emirates described a high prevalence of vitamin D deficiency to insufficiency among the studied patients of 82.5% using a cut-off value of <75 nmol/L (30 ng/ml), despite abundant sunshine in that region.^36^

**Table 4:** Guidelines for Serum 25-Hydroxyvitamin D Status by Various Organisations in the United States of America

	Serum 25(OH)D concentration (ng/ml)		
Vitamin D Status	Endocrine Society^34^	Institute of Medicine^33^	Vitamin D Council^35^
Deficient	<20	<12	0 – 30
Insufficient	21 – 29	12 – 20	31 – 39
Sufficient	**30–100**	**20 – 30**	**40 – 80**
No added benefit		30 – 50	
Toxic/Possible harm	>100	>50	>150

Lack of consensus in optimal vitamin D levels by these organizations is as a result of differences in study design, choice of vitamin D assay methodology and the season or months in which blood samples were taken, geographical latitude of the population under study, and the age range, ethnicity and gender of the study population.^37^ Thus, emphasizing the need for regional (local) and age-specific reference intervals for vitamin D status.

According to US Endocrine Society recommendation, which is also used by the American Geriatric Society, vitamin D concentrations between 21 – 29 ng/ml are designated as vitamin D insufficient, while concentrations ≤ 20 ng/ml are designated as vitamin D deficient^34^ (Table 4). Consequently, 15% of this study participants would have been categorized as being deficient or at least insufficient in vitamin D state using that criteria. But the concentrations of serum calcium of the subjects were within our laboratory's reference interval of 2.15–2.55 mmol/l, which suggested that they have optimal calcium metabolism, and would not support the diagnosis of reduced vitamin D levels. Therefore, the IOM recommendation of desirable vitamin D concentrations > 20ng/ml is more justified among our study population. Despite the differences among the various vitamin D guidelines, they unanimously agree that serum 25(OH)D levels less than 10ng/ml should be avoided at all ages.^38^

Large consensus exists that nearly all elderly individuals need a vitamin D supplement however, there is disagreement among the various guidelines as to the dosage or the optimal concentration of serum 25(OH)D that is adequate. Some guidelines have recommended that for individuals aged 50–70 years, 600IU/day of vitamin D (either D2 or D3) should be taken; while for those over 70years, 800IU/day of vitamin D. 34 Accurate diagnosis of vitamin D deficiency using a locally-derived reference interval for the elderly is important to guide oral vitamin D supplementation, which has become a routine in geriatric medicine. Doses of vitamin D supplements should be increased in elderly people with medical conditions associated with vitamin D deficiency.^39^

We did not observe any significant difference between the serum 25(OH)D concentrations of the male and female participants in this study. This is similar to what was reported by Jungert and Neuhauser-Berthold (2015), which stated that vitamin D status did not differ significantly between men and women.^40^ Gender differences have been suggested for vitamin D status with a higher rate of deficiency occurring in post-menopausal women, increasing the risk of osteoporosis and bone fractures. But our female participants in this study, even though post-menopausal, had higher serum 25(OH levels than the males, similar to Katrinaki et al.^32^

## Limitations

An ideal reference interval study recruits only healthy reference individual. Among the elderly, it was difficult to get the outlined number of subjects without any medical condition or taking medication. However, our questionnaire and screening tests were used to evaluate and exclude overt disease conditions. A larger sample size would have been preferred, but it was challenging to get a large number of ‘healthy’ elderly people. However, we obtained more than the minimum number of recommended reference individuals of 120,^12^ and used the bootstrap method to obtain 1000 samples to give a robust representation. Determination of other bone markers such as parathyroid hormone, bone mineral density; would have been helpful for the bone assessment of the reference individuals, but only serum calcium was assessed, to reduce cost and because, accumulation and level of bone mass can be determined using calcium balance.^33^ Lastly, this study used ELISA method to determine the serum vitamin D levels, rather than mass spectrometry method e.g. LC-MS/MS or GC/MS as is currently used for serum 25(OH)D determination.^41^,^42^ This was due to lack of these methods in our environment, as would be in most developing countries, but the ELISA kit used (Calbiotech ®, USA) reported excellent correlation to LC-MS/MS (n=20, R2 =0.949).^26^

## Conclusion

The reference interval for serum 25(OH)D concentration, which is a measure of vitamin D status, among the selected elderly population in Enugu state, Nigeria was determined to be 21 (90% C.I. 20 - 23) to 93 (90% C.I. 90–98) ng/ml. Although we do not propose the disregard of foreign derived guidelines for serum 25(OH)D status, we suggest that this reference interval be validated in other African elderly populations, which may enable its use in the monitoring of oral supplementation of vitamin D among elderly population in this locale, or similar reference populations.
